# Fuzzy Nonnative Phonolexical Representations Lead to Fuzzy Form-to-Meaning Mappings

**DOI:** 10.3389/fpsyg.2016.01345

**Published:** 2016-09-21

**Authors:** Svetlana V. Cook, Nick B. Pandža, Alia K. Lancaster, Kira Gor

**Affiliations:** ^1^National Foreign Language Center, University of Maryland, College ParkCollege Park, MD, USA; ^2^Graduate Program in Second Language Acquisition, School of Languages, Literatures, and Cultures, University of Maryland, College ParkCollege Park, MD, USA; ^3^Center for Advanced Study of Language, University of Maryland, College ParkCollege Park, MD, USA

**Keywords:** lexical access, phonological representations, form-to-meaning mapping, nonnative auditory perception, Russian

## Abstract

The present paper explores nonnative (L2) phonological encoding of lexical entries and dissociates the difficulties associated with L2 phonological and phonolexical encoding by focusing on similarly sounding L2 words that are not differentiated by difficult phonological contrasts. We test two main claims of the fuzzy lexicon hypothesis: (1) L2 fuzzy phonolexical representations are not fully specified and lack details at both phonological and phonolexical levels of representation (Experiment 1); and (2) fuzzy phonolexical representations can lead to establishing incorrect form-to-meaning mappings (Experiment 2). The Russian-English Translation Judgment Task (Experiment 1, TJT) explores how the degree of phonolexical similarity between a word and its lexical competitor affects lexical access of Russian words. Words with smaller phonolexical distance (e.g., *parent–parrot*) show longer reaction times and lower accuracy compared to words with a larger phonolexical distance (e.g., *parent–parchment*) in lower-proficiency nonnative speakers, and, to a lesser degree, higher-proficiency speakers. This points to a lack of detail in nonnative phonolexical representations necessary for efficient lexical access. The Russian Pseudo-Semantic Priming task (Experiment 2, PSP) addresses the vulnerability of form-to-meaning mappings as a consequence of fuzzy phonolexical representations in L2. We primed the target with a word semantically related to its phonological competitor, or a potentially confusable word. The findings of Experiment 2 extend the results of Experiment 1 that, unlike native speakers, nonnative speakers do not properly encode phonolexical information. As a result, they are prone to access an incorrect lexical representation of a competitor word, as indicated by a slowdown in the judgments to confusable words. The study provides evidence that fuzzy phonolexical representations result in unfaithful form-to-meaning mappings, which lead to retrieval of incorrect semantic content. The results of the study are in line with existing research in support of less detailed L2 phonolexical representations, and extend the findings to show that the fuzziness of phonolexical representations can arise even when confusable words are not differentiated by difficult phonological contrasts.

## Introduction

Current research suggests that second language (L2) learners experience persistent difficulties in auditory perception of non-native speech (for a review, see Gor, 2015). Comprehension of speech by nonnative speakers is typically characterized by a higher propensity for errors and communication breakdowns than in native speakers, and is believed to be more cognitively demanding. The challenges in comprehension are traditionally associated with a difficulty in identifying phonemes that do not exist in the native language (Polivanov, 1932; Sheldon and Strange, 1982; Best, 1994, 1995; Flege et al., 1994, 1996; Flege, 1995; Kuhl and Iverson, 1995; Strange, 1995; Ingram and Park, 1998; Best et al., 2001). A common example is the inability of Japanese learners of English to distinguish between the English /r/ and /l/ phonemes, which are both conflated into a single Japanese phoneme /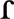/ (Goto, 1971; McClelland et al., 1999; for a recent review, see Cutler, 2015).

Indeed, a reduced ability for phonological categorization of nonnative sounds coupled with unfaithful nonnative *phonological* representations can lead to a breakdown in lexical access (Pallier et al., 1997, 2001; Cutler and Otake, 2004; Weber and Cutler, 2004; Cutler et al., 2006; Broersma, 2012; Diaz et al., 2012). Crucially, a nonnative deficit at the level of phonological representation is only part of the difficulty. Word recognition hinges upon a successful match between the auditory signal and the existing phonological representation of the stored word (Pisoni and Luce, 1987). Therefore, chances of a match are contingent, on the one hand, upon the listener's ability to decode the auditory signal, and, on the other, upon the quality of the *phonolexical* representation, or the phonological representation of the word as a whole (Luce et al., 2000; Chrabaszcz and Gor, 2014). Late L2 learners typically experience deficits in both aspects.

There are two implicit assumptions with respect to the existing relationship between phonology and the lexicon that have recently been subject to scrutiny (see Gor, 2015 for a review). The first assumption is that the acquisition of accurate phonological representations precedes accurate acquisition of lexical knowledge. That is, without establishing distinct phonological representations (or representations of phonemes) it is impossible to establish accurate representations of words containing those phonemes (or phonolexical representations). This is a bottom-up view of lexical acquisition. The second assumption, which is an extension of the first, is that once the nonnative phonological representations are acquired, they can easily transfer into phonolexical representations and contribute to lexical knowledge. This view, and the first assumption in particular, has been challenged by some empirical evidence demonstrating that L2 learners are capable of distinguishing between minimal pairs of words that differ by one phoneme while maintaining unreliable performance on phonological discrimination of those same contrasting phonemes (Weber and Cutler, 2004; Cutler et al., 2006; Escudero et al., 2008; Hayes-Harb and Masuda, 2008). Conversely, as shown by Darcy et al. (2013) in a study of the acquisition of Japanese geminates and German front-rounded and back-rounded vowels by L1 speakers of English, even if the contrast is acquired at a phonological or phonetic level, this knowledge does not necessarily transfer to phonolexical representations. In this study, nonnative participants performed less accurately on nonwords than on words containing the same minimal pair distinction in a lexical decision task. Poor performance on nonwords suggests that L2 speakers were reluctant to reject the nonwords because they were unsure about their phonological composition. This result is in contrast to the results of the phonetic discrimination task, where accuracy was, for the most part, at ceiling. Furthermore, Darcy et al. (2013) extend the original findings of Weber and Cutler (2004) and Cutler et al. (2006), who proposed that the lack of accurate phonological perception does not mandate the lack of a distinction at a phonolexical level; on the contrary, the distinction between two forms can be maintained in the absence of the phonological contrast. It should be noted that if this is the case, the phonological contrast at the phonolexical level is still not target like, and the unfamiliar category tends to be interpreted not as a distinct category in its own right, but rather as a poor exemplar of the familiar (or native) category (see also Escudero et al., 2008; Hayes-Harb and Masuda, 2008). The fact that the distinction between the two phonolexical forms is evident at a lexical, but not at a phonetic level indicates that while there is a phonetic divergence of the two entries from one homophonous form during lexical access, there is no need to postulate a prerequisite of stable phonological representations. More generally, these findings present additional evidence against the “phonology-first,” or bottom-up, approach to acquisition of L2 phonology and suggest an alternative possibility, such that phonological representations evolve together with lexical knowledge, and do not necessarily precede it (Davidson et al., 2007; Dufour et al., 2010; Reinisch et al., 2013).

To complicate the matter of auditory speech perception further, several other factors not directly connected to phonological encoding can interfere with accurate speech perception by L2 speakers, namely those related to lexical knowledge and how this knowledge is represented in the mental lexicon. Some existing research suggests that the organization of the L2 lexicon is qualitatively different from the L1 lexicon in several respects. For example, compared to native speakers, phonological links among words tend to play a much more prominent role in organizing the L2 mental lexicon than semantic links do (Stolz and Tiffany, 1972; Meara, 1978, 1983, 1984; Wolter, 2001; Fitzpatrick, 2006). The main conclusion stemming from these studies is that semantic links between words in the learner's mental lexicon are “fairly tenuous ones, easily overridden by phonological similarities, in a way that is very uncharacteristic of native speakers” (Meara, 1983, p. 31). It is conceivable that L2 learners rely on phonological similarity to make sense of an unknown word. For example, if learners of English hear an unfamiliar word *coffin* without any context to help them figure out the meaning of the word, they may decide that the word is related to the word *cough* or *coffee*.

The representational deficit in lexical knowledge can be quite detrimental to nonnative speech comprehension, especially if it leads to the retrieval of an incorrect word. Because L2 lexical representations are unreliable, and persist into advanced levels of proficiency, L2 learners use additional strategies to resolve phonolexical ambiguities, such as the use of context (Rüschemeyer et al., 2008; Gor, 2014) and morphosyntactic cues (Conrad, 1983; Chrabaszcz and Gor, 2014). When additional cues are unavailable, lexical access can result in retrieval of a non-target word due to increased activation of phonological neighbors and spurious competition (Weber and Cutler, 2004; Broersma and Cutler, 2008, 2011; Broersma, 2012). Words that are less known to the learners are usually associated with greater phonolexical ambiguity and tend to cause error-prone translations in favor of a word phonologically related to the target (Cook and Gor, 2015). This suggests that an unintended word can be accessed as a result of an error in matching auditory input to an existing phonolexical representation, leading to an erroneous form-to-meaning mapping.

While a number of studies have found evidence of separation of pseudo-homophonous phonolexical forms in L2 into distinct, but not necessarily target like, representations (Cutler and Otake, 2004; Broersma and Cutler, 2008, 2011; Broersma, 2012; Darcy et al., 2012, 2013), there is little discussion of the consequences of incorrect lexical access of phonolexically ambiguous words, such as *lock* and *rock*. Indeed, the words used in these studies (except Darcy et al., 2012, 2013) had a clear point of disambiguation, and the ambiguity at the phonetic level created only temporary uncertainty, which was later resolved without affecting the outcome of lexical access. In these studies, the experimental items had little potential for being ambiguous for comprehension beyond the first syllable overlap. For example, for Dutch learners of English, *panda* is only confusable with *pencil* through the presentation of the first syllable, but once the second syllable is reached, the word can be uniquely identified as *panda*, and not *pencil* (Weber and Cutler, 2004).

There is only one study that we are aware of that looked at phonolexical ambiguity in L2 access of the lexical semantics, and form-to-meaning mappings of phonolexically ambiguous words, in particular. Ota et al. (2009) found similar semantic relatedness judgments effects for visually presented pairs of pure homophones (*rock–hard* and *beach–ocean*) and for pairs with pseudo-homophones for English learners of L1 Japanese (*lock–hard*) and L1 Arabic (*peach*–*ocean*). Despite the fact that the study assessed phonolexical ambiguity in words that differed by a phoneme that was perceptually difficult for a particular L1 population (/l/ and /r/ for Japanese, and /p/ and /b/ for Arabic), the study is particularly relevant for the current investigation, since it provides evidence for possible confusion of words' meanings stemming from the lack of detail in their form. The study by Ota and colleagues empirically tested the possibility of erroneous lexical access due to phonolexical ambiguity, and validated the claim that lexical entries for these types of words are resistant to complete separation and are potentially mutually confusable.

The present paper extends the findings of the previous body of research, and tests the *fuzzy lexicon* hypothesis (Cook, 2012; Cook and Gor, 2015). The fuzzy lexicon hypothesis claims that L2 learners operate with *fuzzy*, or low-resolution, phonolexical representations. A fuzzy representation of a word is a mental representation of phonolexical form that does not represent the word as a fixed phonological sequence. Such a representation may leave some phonemes underspecified (e.g., either a final /d/ or /t/) or contain some uncertainty (and ensuing optionality) regarding the exact phonemes and their sequence. Similar to child L1 acquisition, when L2 learners first acquire a word, they represent the word with a purpose to differentiate it from other, similar-sounding words in their lexicon. As in child L1 acquisition, at the initial stages, the representations are approximate, but as the lexicon expands, these fuzzy representations need to be revised. (Note, however, that in both child L1 and adult L2 acquisition, underdifferentiated words may or may not include underdifferentiated phonemes. Such phoneme underdifferentiation would constitute a purely phonological problem, although it will impact word recognition as well.) Crucially for the construct of fuzzy phonolexical representations, lexical underdifferentiation may take place even if there are no phonological problems associated with the word *per se*. As a result, two words may become confusable if they overlap in their form, and their representations are not robust. For example, at the initial stage of acquisition, the word *parent* can be represented as ['pεrә(n)t] with [n] being optional. This type of representation is unstable, because the phonological details of the representation are not fully spelled out. A fuzzy representation has a certain degree of non-targetlike flexibility making it possible to accommodate the input that has contrasting features (both ['pεrәnt] and ['pεrәt]). The learner can successfully operate with this fuzzy representation for the word *parent*, because it is sufficiently detailed to differentiate this word from other words in the mental lexicon. As soon as *parrot* starts to appear in the input, most learners unknowingly continue to map both words *parent* and *parrot* to the same fuzzy representation ['pεrә(n)t] due to fuzziness of the phonolexical representation, which is a match to both words in the input, and to uncertainty about form-to-meaning mappings. With greater vocabulary knowledge and differentiation, at some point learners start to realize that the words *parent* and *parrot* are different both semantically and phonologically. They are forced to revise the existing fuzzy phonolexical representation, and split it into two separate more detailed representations, even if they are still not entirely targetlike.

We make two main assumptions about how fuzzy phonolexical representations function during language use and whether they are of consequence for processing. First and foremost, fuzzy phonolexical representations are not fully specified and lack detail at both the phonological and semantic levels of representation. As a number of studies have successfully demonstrated, including the ones discussed earlier, the presence of an adequate phonological category does not necessarily result in the target like representations of the phoneme at the phonolexical level. To extend this finding further, the fuzzy lexicon hypothesis suggests that in many cases, phonological difficulty associated with the acquisition of L2 phonemes is only one of the factors contributing to the difficulties associated with L2 lexical access. The lack of fidelity in phonolexical representations may or may not have to do with the encoding of a difficult L2 phonological contrast. The main cause of non target like performance is that L2 learners do not know the exact phonological composition of the word that they are trying to access. Fuzzy representations are in many cases episodic, prototype-based, or Gestalt-type representations that allow for mostly reliable access to the correct meaning. Fuzzy phonolexical representations that have a phonological form that is not sufficiently detailed and, consequently, not target like, still make it possible to access the correct meaning.

The second assumption made by the fuzzy lexicon hypothesis concerns incorrect form-to-meaning mappings. The fact that for some time the two representations were merged into a fuzzy representation leads to continued difficulties in correct mapping of the input to the corresponding phonolexical form, and ['pεrәt] can still end up being comprehended as *parent*. Thus, one of the consequences of fuzzy representations is potential confusion in form-to-meaning mappings even when phonolexical fuzziness is partially resolved. This makes the representation unstable, such that the form-to-meaning mapping is still variable and inconsistent, in some cases still resulting in an erroneous match between the auditory input and the phonolexical representation.

The incorrect form-to-meaning mappings are, however, not permanent. As shown by Darcy et al. (2013), L2 proficiency plays an important role in the discrimination of potentially homophonous forms (as was the case with German participants in Experiments 3 and 4), while some phonological contrasts are more resistant to separation (in Experiments 1 and 2 with Japanese participants). The reported lack of separation of potentially homophonous forms (*rock* and *lock*) can result in erratic access to the two potential meanings (*lock* or *rock*). We address these possible scenarios in two auditory experiments with L1 English learners and native speakers of Russian.

### The present study

This study aims to further explore how confusability at the phonolexical level affects nonnative lexical access. To our knowledge, the present study is among the first to extend the focus of the research beyond the lexical encoding of difficult phonological contrasts and to deal with phonolexical representations in terms of their global similarity in phonolexical form (as in *parent–parrot*, for example). While difficult phonological contrasts and global similarity in phonolexical form both influence lexical processing, we seek to assess the quality of phonolexical representations on their own right by eliminating the need to encode problematic L2 phonemes.

In Experiment 1, we explore the first assumption made by the fuzzy lexicon hypothesis, which claims that at early stages of L2 acquisition, the phonolexical form of a new word is stored without a detailed specification. As a result, it is confusable with similar sounding words. The present experiment makes an assumption that the likelihood that a word will be confused with another word is determined by the degree of phonological overlap between the two words. In a Translation Judgment Task (TJT) with aurally presented Russian words, we explore how varying degrees of similarity affect lexical access. We operationalize this similarity between competing phonolexical forms as phonological Levenshtein Distance (LD), with higher LD indicating less phonological overlap. Three groups of participants completed the task: Advanced and Superior L2 learners of Russian, and a group of native Russian speaker controls. In critical trials, actual Russian word primes were replaced by a competitor Russian word with a similar phonolexical form. We predict that L2 learners will be less accurate than Native speakers in judging the translation of words that differ from the competitor in only one or two phonemes. Since the phonolexical form of L2 words is represented coarsely and without fine detail, during the matching procedure, fine differences between the auditory stimuli and the available representations stored in the mental lexicon will be overlooked. Alternatively, they may be discounted as allowable variation due to speaker or pronunciation differences. Further, the lower-proficiency Advanced L2 group will show less sensitivity to the differences between the target word and its competitor, because lower-proficiency L2 speakers represent words more “holistically” than the higher-proficiency Superior L2 speakers. As a consequence, for the lower-proficiency L2 group, the confusability effect for a mismatch in one phoneme will be similar to the effect for a mismatch in two phonemes. Unlike the Advanced group, the Superior group will have a greater sensitivity to the increase in the LD between the competitors, because they operate with a higher-definition phonolexical representation, which gives them more chances to detect the mismatch.

Experiment 2 addresses the second assumption made by the fuzzy lexicon hypothesis and assesses the vulnerability of form-to-meaning mappings as a consequence of fuzzy phonolexical representations in L2. In a modification of a semantic priming experiment (a Pseudo-Semantic Priming task, PSP) we primed the target with a word semantically related to its competitor, but not to the target itself. Learners heard the word *коРовА* /karova/ “cow” as the prime and then *молоток* /malatok/ “hammer” as the target. We hypothesized that they would be biased to think that they had heard a word they knew and expected—*молоток* /malako/ “milk.” Indeed, the two words sound very much alike, and the L2 learner temporarily identifies the word *молоток* /malatok/ “hammer” as the closest phonological match *молоко* /malako/ “milk,” which is semantically related to “cow.” Thus, the target word is confused with another one based on the similarity of their phonological forms. The predictions are that both a native and nonnative listener will expect to hear “milk” after they hear “cow.” At a point in time when they realize that they hear “hammer” instead of the onset-matched “milk,” a native speaker will quickly recover from the unmet expectation, while a nonnative speaker will be slower in recovering from the semantic “garden path” created by the prime and the target with a highly expected onset. If L2 learners show an increase in the processing time for a pseudo-target (“hammer”), this will be an indication that some confusion at the level of phonolexical representations has taken place. This scenario is only possible if neither of the words has a phonological representation that is detailed enough, or if there is an imbalance between them in terms of frequency and, thus, availability for efficient L2 lexical access. Sekine (2006) reports that lower-frequency L2 words have a tendency to be identified as similar-sounding higher-frequency words during auditory perception. While these results can be also explained by the acquisitional sequence, where words of higher frequency are learned before words of lower frequency, the critical difference between the two confusable words remains—one has a more detailed representation than the other. The pattern of substitution will be to replace a lesser-known word with a better-known word. It is also possible that if the learner is not able to make a distinction between the forms of two similar-sounding words in the mental lexicon, both phonological forms will be loosely linked to the respective meanings, and can be swapped in lexical access. This assumption aligns with the fuzzy lexicon hypothesis: under certain circumstances, fuzzy phonological representations can activate the lexical meaning of the competitor, and as a result the wrong lexical meaning could be accessed. This is exactly the effect that the pseudo-priming experiment is designed to produce. If our assumption is true, then learners will tend to confuse the pseudo-related target with the actual semantically related word, resulting in less accurate judgment of lexical acceptability and slower reaction time in making the judgment.

## Experiment 1: translation judgment task (TJT)

### Method

#### Participants

Thirty-two native speakers of Russian (22 female) and 52 adult American learners of Russian (33 female) participated in Experiment 1. Table 1 displays the language background and demographic information of the speaker groups. Native speakers of Russian on average spent 18.9 years in the classroom learning English (*SD* = 4.1) and began learning English at an average age of 8.3 (*SD* = 3.0). Their self-rated English proficiency was on average 8.13 (*SD* = 1.8) for grammar, 8.34 (*SD* = 1.43) for speaking, 8.97 (*SD* = 1.12) for listening, and 9.13 (*SD* = 1.07) for reading on a ten-point Likert scale (0—“no proficiency” to 10—“native-like command”).

**Table 1:** **Experiment 1 (TJT) language background and demographic information by participant group**.

**Group**	**Advanced (*n* = 21)**	**Superior (*n* = 31)**	**Native (*n* = 32)**
	***Mean (SD)***	***Mean (SD)***	***Mean (SD)***
Age	27.5 (4.1)	29.5 (8.0)	30.7 (9.0)
Age of acquisition	18.1 (2.8)	19.0 (3.1)	–
Classroom instruction	3.3 (2.1)	3.1 (2.0)	–
Immersion experience	1.6 (1.8)	2.8 (1.6)	–

*All variables are measured in years*.

All nonnative participants prior to participation were pre-tested with a standard test of oral proficiency, a formal Oral Proficiency Interview (OPI), which assigned them a proficiency level in Russian on the Interagency Language Roundtable (ILR) scale widely used in the USA for government testing. Based on the OPI scores, L2 participants were subdivided into two proficiency groups matched to the ILR levels 2 and 2+ (*n* = 21), and 3 and 3+ (*n* = 31), with higher scores indicating higher proficiency levels. Respectively, these group levels correspond to Advanced and Superior oral proficiency on the American Council on the Teaching of Foreign Languages (ACTFL) academic scale. All participants completed a language background questionnaire.

#### Materials

Russian words were selected from two frequency ranges—high (HF, ~130–500 instances per million) and low (LF, ~30–100 instances per million). The experimental set included words from different grammatical categories, but the majority belonged to the noun, verb, and adjective classes. The stimuli varied in phonological length (4–10 phonemes) and syllabic length (1–4 syllables). The experimental trials were counterbalanced across the two presentation lists. Since the same target appeared in matched and unmatched conditions on different presentation lists, lexical parameters of the items were naturally balanced across lists.

Each participant completed a total of 162 trials, each with an auditorily presented Russian word followed by a visually presented English word. In half of the trials, the Russian and English words matched (i.e., the Russian and English words were translations of one another), while in the other half of the trials the words mismatched (i.e., the Russian and English words were not translations of one another). For instance, one matched trial began with the auditory presentation of the Russian word *молоток* /malatok/ “hammer,” followed by the visual presentation of the English word HAMMER. Presentation lists were balanced along the matching condition, such that if one target word appeared in a matched trial in List A, the same target appeared in a mismatched trial in List B.

In addition to the matching manipulation, the Russian words in the mismatch trials were manipulated using Levenshtein Distance (LD). LD is the measure used to calculate the degree of overlap between two phonological forms. It represents the “distance” between two word forms as measured by the number of replacements, additions, and deletions needed to generate one from the other (Levenshtein, 1966). For example, the Russian words *молоток* /malatok/ “hammer,” and *молоко* /malako/ “milk” have an LD of two. By two changes, replacing the /t/ in /malatok/ with a /k/ and removing the final /k/, /malatok/ becomes /malako/. The psychological reality of LD and similar metrics has been demonstrated in various psycholinguistic tasks (e.g., Beijering et al., 2008; Yarkoni et al., 2008). In the mismatch trials, the Russian word was similar in form to the actual Russian translation of the English word presented. Thus, in these trials, the Russian word acted as a competitor to the actual translation. For instance, in one competitor mismatch trial /malako/ “milk” was followed by HAMMER. Since /malako/ “milk” and the actual Russian translation of “hammer,” /malatok/, have an LD of two, we expected participants to respond differently to these trials than to non-competitor mismatch trials (e.g., /zvezda/ “star”—BASEMENT). The experiment contained 54 non-competitor mismatch trials in which the Russian word heard and the actual Russian translation were not phonolexically similar. Within the competitor mismatch trials, the LD between the Russian word presented and the actual Russian translation ranged from 1 to 5.

#### Procedure

After completing a prescreening, which included the language history questionnaire, potential participants were invited to participate in the experiment. Participants completed the study remotely using DMDX testing software (Forster and Forster, 2003). Consent form and procedures were approved by the University of Maryland Institutional Review Board. The participants were instructed to take the test individually on a computer with headphones in a quiet room. The TJT was a part of a larger set of tasks not reported here. This test took ~20 min to complete, and all participants were paid upon completion of the study.

The materials for both experiments were digitally recorded by the same female native speaker of Russian in a sound-attenuated booth. Recordings were broadcast wave files (16 bit/48 kHz), made on a *Zoom H4n* digital audio recorder. The speaker read the items one by one in a clear citation style. Three or more recordings of each item were made, and the best-sounding token was chosen and included in the test stimuli. The sounds were digitally processed in Praat (Boersma and Weenink, 2013). Each individual token was extracted from the original recording at a zero-crossing boundary. Upon extraction, all stimuli were normalized for intensity.

A single trial consisted of a fixation cross, presented in the center of the screen for 250 ms, followed by a blank screen for 50 ms; then the auditory Russian prime was presented (the screen remained blank throughout the presentation of the audio file). At the offset of the prime the screen continued to remain blank for the duration of the inter-stimulus interval (ISI) for 1500 ms; a visually presented English target word immediately followed (centered on the screen, typeset Calibri, size 12, in bold, all upper-case letters). The target remained on the screen until the response was made or until the trial timed out (4000 ms from the onset of the visually-presented target). If no response was given when the timeout was reached, the next trial was advanced without a button press. Each trial was followed by a 1000 ms inter-trial interval (ITI). Participants were instructed to decide whether the two words were translation equivalents or not by pressing the appropriate button on the computer keyboard (right Control key for “YES” and left Control key for “NO”). Accuracy and reaction time (RT) from the onset of the visual target were digitally recorded. Participants completed nine practice trials before beginning the experimental trials. All stimuli were presented in 5 blocks (4 blocks with 35 and 1 block with 22 trials each), with opportunities for the participants to take self-paced breaks between the experimental blocks. Except for the practice trials, there was no feedback on accuracy provided to the participants.

### Results

To model our data, we employed multilevel modeling (MLMs, or mixed-effects models) because of several advantages the method yields over traditional multiple regression or ANOVA methods: (1) by-subject and by-item analyses can be done simultaneously, so as to generalize across people and items within a single analysis; (2) each individual trial is included in the analysis rather than averaging across multiple trials to obtain a single value for each participant; and (3) it properly models the multilevel structure of the data (e.g., trial-level variables such as word frequency vs. subject-level variables such as language proficiency) and is therefore not subject to the assumption of independence of observations as are multiple regression or ANOVA (Baayen et al., 2008; Linck and Cunnings, 2015).

The multilevel models we report here were conducted with the lme4 package version 1.1-9 (Bates et al., 2015) in R version 3.2.0 (R Core Team, 2015) for logistic and linear multilevel modeling. Logistic MLMs for accuracy analyses were run using the “bobyqa” optimizer. In the RT analyses, correct responses were trimmed to exclude RTs lower than 300 ms because these reflect RTs that are too fast for normal processing, after which responses with long RTs were excluded if they exceeded a three standard deviation by-participant cutoff. Linear MLMs for RTs were reported using restricted maximum likelihood estimation, as full maximum likelihood underestimates the standard errors of the estimates. Due to the ongoing debate in calculating *p*-values for linear MLMs, only *t*-values are provided in lme4 output, so |*t*| > 1.65 is considered marginal (*p* < 0.10), and |*t*| > 2.00 is considered significant at *p* < 0.05 (Gelman and Hill, 2007). All models were run as forced entry models for fixed effects and cross-classified subject and item random intercepts, and random slopes were tested one-by-one via likelihood ratio tests; only random slopes that significantly improved model fit and resulted in converging models were retained (Baayen, 2008; Baayen et al., 2008).

#### Accuracy

Due to the low number of stimuli at LD 5, those items were excluded from further analysis (1.5% of observations). Two more items were excluded from further analysis (1.5% of observations) due to technical issues. Accuracy results were then submitted to a logistic multilevel model (Table 2). The dependent variable was accuracy (0, 1); fixed effects included Condition (dummy-coded: Match, Competitor Mismatch, Non-Competitor Mismatch), Russian auditory prime match Frequency (log-transformed and *z*-scored), phonological LD between the competitor and the auditory match (LD 1–4; centered on LD 1, which is an LD of 1 phoneme), and Proficiency (dummy-coded as Advanced: ILR scale 2 and 2+, Superior: ILR 3 and 3+, and Native), as well as all two- and three-way interactions except those involving LD and Condition, as LD is only relevant to the Competitor Mismatch condition. Native speakers and the Competitor Mismatch condition were baseline; thus all significant effects in the model are interpreted with respect to this baseline (e.g., a significant effect for Advanced signifies the group is significantly different than the Native group). Note that a logistic MLM is not modeling mean accuracy but the probability of a correct or an incorrect response on an item given the predictors in the model.

**Table 2:** **Experiment 1 (TJT) results of logistic multilevel modeling for Accuracy**.

**Fixed effects**	***b***	**exp(*b*)**	***SE***	***p* value**
Intercept (Native/Competitors/LD 1)	3.85	46.99	0.29	< 0.001^*^
Group:			
Advanced	−1.59	0.20	0.35	< 0.001^*^
Superior	−1.73	0.18	0.33	< 0.001^*^
Russian target freq (Native/LD 1)	−0.69	0.50	0.22	< 0.01^*^
Freq × Advanced	0.39	1.48	0.17	< 0.01^*^
Freq × Superior	0.29	1.34	0.17	0.08^∧^
Levenshtein distance (Native)	0.38	1.46	0.24	0.11
LD × Advanced	−0.12	0.89	0.28	0.67
LD × Superior	0.39	1.48	0.28	0.17
LD × Freq (Native)	0.25	1.28	0.17	0.15
LD × Freq × Advanced	0.34	1.40	0.14	0.02^*^
LD × Freq × Superior	0.27	1.31	0.15	0.07^∧^
Condition:				
Match (Native)	0.31	1.36	0.47	0.50
Match × Advanced	0.85	2.34	0.56	0.13
Match × Superior	2.09	8.08	0.54	< 0.001^*^
Match × Freq	1.57	4.81	0.29	< 0.001^*^
Non-competitor Mismatch (Native)	2.87	17.64	0.55	< 0.001^*^
NCM × Advanced	0.97	2.64	1.42	0.16
NCM × Superior	2.08	8.00	0.67	< 0.01^*^
NCM × Freq	1.17	3.22	0.32	< 0.001^*^
**Random effects**	**Variance**	***SD***	**Correlation**	
Intercepts | Subject	0.18	0.43		
Intercepts | Item	2.31	1.52		
Advanced | Item	1.21	1.10	−0.32	
Superior | Item	1.04	1.02	−0.25 0.97	

* Significant at p < 0.05;

∧ *Marginal at p < 0.10. Covariates are shaded in gray*.

The model intercept indicates that Competitor Mismatch trials are more likely than not to be correctly identified as incorrect translations by Native speakers, although Advanced (*b* = −1.59, *SE* = 0.35, *p* < 0.001) and Superior (*b* = −1.73, *SE* = 0.33, *p* < 0.001) are both about five times less likely to correctly respond to LD 1 competitors (but note high accuracy overall in Table 3).

**Table 3:** **Mean accuracy to Russian match trials, non-competitor mismatch trials, and competitor trials of different Levenshtein distance split by frequency for both native and nonnative speakers in Experiment 1 (TJT)**.

**Group**	**Frequency**	**Match**	**Non-competitor mismatch**	**Competitor mismatch (levenshtein distance)**			
				**1**	**2**	**3**	**4**
**NATIVE**							
	High	0.96 (0.19)	0.99 (0.09)	0.96 (0.20)	0.92 (0.20)	0.98 (0.14)	0.99 (0.11)
	Low	0.91 (0.29)	0.99 (0.09)	0.97 (0.16)	0.98 (0.15)	0.99 (0.12)	1.00 (0.00)
**SUPERIOR**							
	High	0.95 (0.21)	0.99 (0.09)	0.77 (0.42)	0.87 (0.34)	0.96 (0.19)	0.99 (0.10)
	Low	0.91 (0.28)	0.99 (0.09)	0.89 (0.31)	0.90 (0.30)	0.97 (0.17)	1.00 (0.00)
**ADVANCED**							
	High	0.93 (0.25)	0.99 (0.10)	0.83 (0.38)	0.93 (0.26)	0.91 (0.29)	1.00 (0.00)
	Low	0.86 (0.34)	0.98 (0.13)	0.87 (0.33)	0.88 (0.32)	0.91 (0.29)	0.78 (0.43)

*Smaller Levenshtein distance indicates greater phonological similarity*.

All three groups show an inverse effect of frequency on LD 1 trials, such that performance is worse as competitor frequency increases. Specifically, the Native group shows the strongest disadvantage to frequency at LD 1 (*b* = −0.69, *SE* = 0.22, *p* < 0.01), while the Superior group shows a trend for a weaker effect (*b* = 0.29, *SE* = 0.17, *p* = 0.08) and the Advanced group shows a significantly weaker effect (*b* = 0.39, *SE* = 0.17, *p* < 0.01). Table 3 lists the average accuracy as LD increases by speaker group, with LD clearly affecting nonnative speakers but not native speakers. Covariate interactions indicate that for Match and Non-Competitor Mismatch conditions, there is a strong canonical frequency effect such that, as frequency of the Russian word they heard increases, participants are more likely to correctly respond to the English translation.

No group shows a significant effect of LD independent of frequency (all *p*s > 0.10), and the Native group does not show any effect of LD with increasing frequency. However, the Advanced group does show a positive effect of increasing LD with increasing frequency (*b* = 0.34, *SE* = 0.14, *p* = 0.02) such that, as the frequency of the competitor word increases, participants are more accurate the less phonological overlap the Russian word has with the correct Russian translation. The Superior group also shows a similar positive trend (*b* = 0.27, *SE* = 0.15, *p* = 0.07). Taken together with the patterns observed in Table 3 these effects suggest that the high frequency trials are driving the LD effect in L2 learners, and that accuracy increases with the increase in the LD between the incorrect Russian competitor and the correct Russian translation of the target.

#### Reaction time

RT results for correct responses were trimmed as described above (eliminating 0.7% of observations) and submitted to a linear multilevel model (Table 4). All fixed effects, including interactions and baselines were identical to those in the logistic MLM for the accuracy data above. The random effects structure differed in that additional random slopes significantly improved the fit of this model, likely due to the large variability in RT whereas accuracy was largely high and near-ceiling for some conditions.

**Table 4:** **Experiment 1 (TJT) results of linear multilevel modeling for RT**.

**Fixed effects**	***b***	***SE***	***t* value**
Intercept (Native/Competitors/LD 1)	6.66	0.04	167.91^*^
Group:			
Advanced	0.04	0.06	0.71
Superior	−0.001	0.06	−0.01
Russian target frequency (Native/LD 1)	0.03	0.01	2.44^*^
Freq × Advanced	−0.003	0.01	−0.26
Freq × Superior	0.002	0.01	0.21
Levenshtein distance (Native)	0.002	0.01	0.16
LD × Advanced	−0.03	0.02	−1.68^∧^
LD × Superior	−0.01	0.02	−0.96
LD × Freq (Native)	−0.03	0.01	−3.83^*^
LD × Freq × Advanced	−0.002	0.01	−0.31
LD × Freq × Superior	−0.00001	0.01	0.00
Condition:			
Match (Native)	−0.19	0.03	−6.60^*^
Match × Advanced	−0.16	0.05	−3.48^*^
Match × Superior	−0.10	0.04	−2.50^*^
Match × Freq	−0.10	0.02	−5.89^*^
Non-competitor mismatch (Native)	−0.07	0.03	−2.58^*^
NCM × Advanced	−0.16	0.04	−3.92^*^
NCM × Superior	−0.11	0.04	−3.12^*^
NCM × Freq	−0.07	0.02	−4.21^*^
**Random effects**	**Variance**	***SD***	**Correlation**
Intercept | Subject	0.04	0.21	
LD | Subject	< 0.001	0.02	−0.41
Frequency | Subject	< 0.001	0.01	−0.51 −0.42
Match | Subject	0.01	0.10	−0.49 0.51 0.35
NCM | Subject	< 0.01	0.06	−0.67 0.87 0.08 0.78
Intercept | Item	0.01	0.11	
Advanced | Item	0.01	0.09	−0.20
Superior | Item	0.01	0.08	−0.09 0.65
Residual	0.09	0.30	

* Significant at p < 0.05;

∧ *Marginal at p < 0.10. Covariates are shaded in gray*.

**Figure 1:**
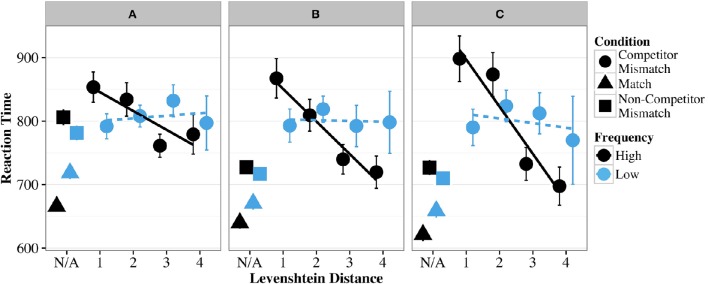
**Mean RTs of match trials, non-competitor mismatch trials, and competitor mismatch trials of different Levenshtein distances for words of high and low frequency in Experiment 1 (TJT)**. Lines indicate linear regression lines of best fit for competitor mismatch targets of high and low frequency. **(A)** Represents native speakers, while **(B)** represents nonnative speakers of Superior proficiency, and **(C)** represents nonnative speakers of advanced proficiency.

As Figure 1 suggests (also see Supplementary Material for raw values), the Competitor Mismatch trials at LD 1 are more slowly responded to than match and Non-Competitor Mismatch trials to varying degrees for all groups.

As in the accuracy data, all three groups show an inverse effect of frequency on LD 1 trials, such that performance slows as competitor frequency increases; however, unlike the accuracy data, Native (*b* = 0.03, *SE* = 0.01, *t* = 2.44), Superior (*b* = 0.002, *SE* = 0.01, *t* = 0.21), and Advanced groups (*b* = −0.003, *SE* = 0.01, *t* = −0.26) all show an effect in RT as the effect for Natives was significant and the interaction terms for Superior and Advanced are not statistically different from that effect. Covariate interactions indicate (as they did for the accuracy data) that for Match and Non-Competitor Mismatch conditions, there is a strong canonical frequency effect such that, as frequency of the Russian word they heard increases, participants are faster to respond correctly to the English translation.

None of the groups shows a significant effect of LD independent of frequency; however, the Advanced group shows a marginal effect (*b* = −0.03, *SE* = 0.02, *t* = −1.68) for faster responses as LD increases (i.e., phonological overlap decreases). Unlike in the accuracy data, the Native group here does show a strong effect of LD with increasing frequency (*b* = −0.03, *SE* = 0.01, *t* = −3.83), as do the Superior (*b* = −0.001, *SE* = 0.01, *t* = 0.00) and the Advanced groups (*b* = −0.002, *SE* = 0.01, *t* = −0.31), whose performance is not significantly different from the Native group. Thus, as the frequency of the competitor word increases, participants respond more quickly the less phonological overlap the Russian word has with the correct Russian translation. Taken together with the patterns observed in Figure 1, these effects suggest that the high frequency trials are driving the LD effect for all groups (with the possible exception of the Advanced group), and that speed increases with the increase in the LD value, or as the phonological similarity between the words decreases.

In order to evaluate the strength of the confusability effect as a function of phonolexical distance independently of the frequency manipulation, we fitted an additional model to the high frequency RT data alone. Only the Advanced group show an independent effect of phonolexical distance (*b* = −0.05, *SE* = 0.02, *t* = −2.28), evidenced by a greater slowdown in lexical access with confusable words that differed from the competitor in one or two phonemes (LD 1 and LD 2) compared to the words that differed in two or three phonemes (LD 3 and LD 4). In contrast, neither Native nor Superior group demonstrates a significant effect of phonolexical distance (*b* = −0.02, *SE* = 0.01, *t* = −1.82 and *b* = −0.02, *SE* =.02, *t* = −1.17 for the Native and Superior groups, respectively).

### Discussion

The results of Experiment 1 revealed that the nonnative ability to access the target word reliably is associated with the degree of phonolexical similarity (more dissimilar words are easier to tell apart), and improves with higher proficiency. A reduced accuracy rate in the Advanced group in rejecting the incorrect translations with smaller phonological differences (LD 1 and LD 2) indicates that the Advanced participants are willing to accept auditory forms with a much greater phonological variability than the Superior or Native participants. Lower-proficiency L2 learners require a greater degree of difference between the words with potentially confusable phonolexical representations to differentiate among them and efficiently establish a correct match. As proficiency increases, this constraint is no longer at play and even small deviations from the target form are detected. Thus, we have found support that the effect in the Advanced group is driven by the reduced ability of the learner to match the auditory stimulus to the available phonolexical representations.

The RT data further confirm the picture. All groups have demonstrated an effect of phonolexical distance, which interacted with frequency, such that an increase in competitor frequency resulted in a slowdown as the number of differentiating phonemes decreased. Words with competitors only minimally different from the intended target incurred the greatest processing costs.

In sum, the results of Experiment 1 suggest that unlike Native speakers, lower-proficiency learners do not properly encode the phonolexical information, and are thereby prone to access the incorrect lexical representation of a lexical competitor.

## Experiment 2: pseudo-semantic priming (PSP)

### Method

#### Participants

Forty-seven adult American learners of Russian (9 female) and 20 adult native Russian controls (11 female) participated in Experiment 2. All nonnative participants were assigned to one of two proficiency levels: Intermediate or Advanced. Table 5 displays the language background and demographic information of each speaker group. Participants were recruited throughout the United States at universities with Russian Language Programs. As seen in Table 5, the Advanced learner group had spent more time in Russian-speaking countries than the Intermediate group [*t*_(43)_ = 9.73, *p* < 0.001]. However, the L2 groups are similar in duration of classroom instruction due to the structure of the program in which the Advanced students were enrolled [*t*_(31.1)_ = 0.17, *p* = 0.566]. That is, many Advanced learners were in a program which did not require extensive classroom instruction before immersion in a Russian-speaking country.

**Table 5:** **Experiment 2 (PSP) language background and demographic information by participant group**.

**Group**	**Intermediate (*n* = 20)**	**Advanced (*n* = 27)**	**Native (*n* = 20)**
	***Mean (SD)***	***Mean (SD)***	***Mean (SD)***
Age	24.3 (5.2)	23.9 (3.6)	23.0 (4.2)
Age of acquisition	21.0 (5.9)	20.2 (4.5)	–
Classroom instruction	2.8 (2.0)	2.4 (1.5)	–
Immersion experience	0.4 (0.4)	1.7 (0.5)	–

*All variables are measured in years*.

The determination of proficiency assignment was predominantly based on the results of a *C*-test (see Section Materials for details); however, other background information was also an important factor, such as length of study and length of immersion. In addition, the participants provided self-assessment data on their abilities in Speaking, Writing, Pronunciation, and their estimate of their L2 lexicon size. All these factors were taken into account for the group assignment. For example, if prospective participants had a significant immersion experience and high self-assessment scores, but had a borderline score on the *C*-test, they were assigned to the Advanced group.

#### Materials

In order to prescreen the participants in terms of their level of Russian language proficiency, a *C*-test was constructed based on the story “Modern day Mowgli,” which was adopted for testing purposes from a Russian language textbook (Niznik et al., 2009). A *C*-test is assumed to be a reliable measure of global language proficiency (Eckes and Grotjahn, 2006) and can also be successfully used in vocabulary research as a measure of vocabulary size (Singleton and Little, 1991; Singleton and Singleton, 1998; Singleton, 1999). According to the specification of the test, the first sentence of the text remained unchanged, and starting with the second sentence, every other word was partially deleted. The deletion was done according to the prescribed methodology: if the word has an even number of letters, the split is done in the middle, and the beginning half of the word is presented to the test-taker; if the word has an uneven number of letters, then the beginning half is preserved plus one additional letter, and this combination is presented to the test-taker. This process led to 40 partially deleted words. The scoring was done on a 3-point scoring scale for each testing item. Three points were assigned for a correct answer; two points were assigned for a correct vocabulary item, but in an incorrect form, resulting from an incorrect inflection (number, person, gender, tense, and mood errors); one point was assigned for a correct vocabulary item in a default form, i.e., uninflected; and zero points were assigned for an incomplete or incorrect vocabulary item. The ceiling accuracy score was 120 points (40 × 3 points per item). All of the Advanced participants scored above a 100 point mark on the *C*-test (*M* = 107.14, *SD* = 4.11), while the participants in the Intermediate group showed much greater variability, with scores distributed over a larger range (*M* = 76.79, *SD* = 17.14).

In Experiment 2 participants were required to listen to two Russian words and indicate if the second word (target) was a real Russian word. There were 320 trials in this experiment, half of which (160) included real words and the other half included nonword targets. Nonwords were created from real Russian words by manipulating the first syllable; primes were always real words. Frequency was matched within prime-target pairs (high, low frequency); other parameters (e.g., number of syllables, length in phonemes) were balanced across conditions. Due to proficiency limitations, the Intermediate group was only exposed to a subset of the experimental materials, those in the high-frequency condition, therefore, the number of experimental trials for this group was reduced (160 overall instead of 320).

For the experiment we created 40 pairs that were related semantically, 40 pairs for the pseudo-semantic condition, 20 pairs for the unrelated (control) condition, and 100 distractor trials. Words in the unrelated trials were matched to the words in the critical conditions in frequency and length in phonemes. Real word prime-target pairs were created for the semantic priming condition (e.g., коРовА /karova/ “cow”—молоко /malako/ “milk”). Then a matching pseudo-semantic target was selected for each prime, appearing in the semantic condition (коРовА /karova/ “cow”—молоток /malatok/ “hammer”). The words for the pseudo-semantic condition were selected based on their phonological similarity to the semantically related target and were always lower in frequency, but still within the targeted frequency band (high or low). Keeping in mind the frequency split (high, low), the materials were also constructed by using words that were moderately known to the participants. That is, known well enough for the lexical meaning to be accessed, but not well enough to accurately access the correct phonological representation in the mental lexicon. Pseudo-semantic pairs were pilot-tested on two Russian language learners prior to the study. The pilot-testers were not participants of the present study. Items that performed the best were retained for the use in the experiment.

The semantic and pseudo-semantic trials were balanced across two presentation lists, and during the experiment, each participant heard each prime only once, either in the semantic or in the pseudo-semantic condition. For instance, /malatok/ was heard if the priming pair is a pseudo-semantically related pair, or /malako/ if the target was a true semantically related word. Among the 20 native and 20 intermediate speakers, presentation list type (A, B) was evenly split. Due to the uneven number of advanced speakers, 14 heard list A while 13 heard list B.

#### Procedure

After completing a prescreening, which included the language history questionnaire and *C*-test, potential participants were invited to participate in the experiment as part of the Intermediate or the Advanced group. There were two ways that participants could complete the study—in person or remotely. The same testing software, DMDX (Forster and Forster, 2003), and delivery sequence was used in both methods. Consent form and procedures were approved by the University of Maryland Institutional Review Board. Each participant took the test individually on a computer with headphones in a quiet room. The results of the PSP presented here were part of a larger set of tasks not reported here. The experiment took ~30 min to complete. All participants were paid upon completion of the study.

A single trial consisted of a sequence of two aurally presented lexical items. Each trial started with a 300 ms pre-stimulus interval, then the audio prime was played in its entirety. The prime was followed by an ISI of 300 ms, after which the audio target was presented. Auditory stimuli were always played in their entirety, and subjects were given 4000 ms from the onset of target presentation to respond. Participants were instructed to decide whether the second word (the target) is a real Russian word or not by pressing the appropriate button on the computer keyboard (right Control key for “YES” and left Control key for “NO”). Each trial was followed by a 600 ms ITI. Accuracy and RT from the onset of the auditory target were digitally recorded. If no response was given after 4000 ms, the next trial was advanced without a button press. RT and accuracy were digitally recorded. Participants completed 10 practice trials before beginning the experimental trials. All experimental stimuli were presented in 8 blocks of 40 trials each with opportunities for participants to take self-paced breaks between the experimental blocks. Throughout the experiment, participants received feedback on the accuracy and speed of their responses to motivate optimal performance.

### Results

The following logistic and linear multilevel models were conducted in the same manner as described in Experiment 1, with the exception of the cross-classified subject and item structure. The random effects structure for both models consisted of random intercepts by subject crossed by random intercepts by prime word nested within random intercepts by unique prime-target item pair, due to the nature of how the stimuli were constructed.

#### Accuracy

Accuracy results were submitted to a logistic multilevel model (Table 6). The dependent variable was accuracy (0, 1); fixed effects included Condition (dummy-coded: Control, Semantic Priming, and Pseudo Priming), Word Pair Frequency (HF, LF), and Group (dummy-coded: Intermediate, Advanced, and Native), and all two- and three-way interactions thereof. The model baseline was high frequency control trials for the Native group, and so all effects are to be interpreted with respect to this baseline. To help visualize the effects presented in the model, a simplified characterization of the data as cell means is presented in Table 7.

**Table 6:** **Experiment 2 (PSP) results of logistic multilevel modeling for Accuracy**.

**Fixed effects**	***b***	**exp(*b*)**	***SE***	***p* value**
Intercept (Native/Control/HF)	3.51	33.45	0.34	< 0.001^*^
Group:				
Intermediate	−1.23	0.29	0.35	< 0.001^*^
Advanced	0.35	1.42	0.38	0.51
Condition:				
Semantic Priming (Native/HF)	2.56	12.94	1.08	0.02^*^
Semantic × Intermediate	−4.45	0.01	1.07	< 0.001^*^
Semantic × Advanced	−1.85	0.16	1.14	0.10
Pseudo Priming (Native/HF)	−1.17	0.31	0.45	< 0.01^*^
Pseudo × Intermediate	−0.72	0.49	0.41	0.08^∧^
Pseudo × Advanced	−0.80	0.45	0.44	0.07^∧^
Low frequency (Native)	−0.02	0.98	0.46	0.96
LF × Advanced	−1.05	0.35	0.49	0.03^*^
LF × Semantic (Native)	−0.15	0.86	1.53	0.92
LF × Semantic × Advanced	−0.18	0.84	1.56	0.91
LF × Pseudo (Native)	−0.79	0.45	0.62	0.20
LF × Pseudo × Advanced	1.18	3.25	0.58	0.04^*^
**Random effects**	**Variance**	***SD***	**Correlation**	
Intercept | Subject	0.31	0.56		
Low frequency | Subject	0.40	0.64	−0.78	
Intercept | Prime	0.56	0.75		
Intercept | Prime/Item Pair	0.56	0.75		

* Significant at p < 0.05;

∧ *Marginal at p < 0.10*.

**Table 7:** **Mean accuracy to Russian pseudo-semantic priming trials, semantic trials, and control trials split by frequency for both native and nonnative speakers in Experiment 2 (PSP)**.

**Group**	**Frequency**	**Pseudo-Semantic Priming**	**Semantic Priming**	**Unrelated Control**
**NATIVE**				
	High	0.87 (0.34)	1.00 (0.07)	0.95 (0.21)
	Low	0.78 (0.42)	1.00 (0.07)	0.95 (0.22)
**ADVANCED**				
	High	0.80 (0.40)	0.98 (0.15)	0.96 (0.19)
	Low	0.71 (0.45)	0.93 (0.26)	0.90 (0.30)
**INTERMEDIATE**				
	High	0.56 (0.50)	0.57 (0.50)	0.86 (0.34)

*The Intermediate group was not exposed to low frequency trials*.

On HF Control trials, the Native and Advanced group (the latter not statistically different from the Native group) perform more accurately than the Intermediate group (*b* = −1.23, *SE* = 0.35, *p* < 0.001).

For HF semantic priming trials, the Native and Advanced groups are significantly more accurate compared to control trials, showing a strong semantic priming effect. However, the effect for the Intermediate group (*b* = −4.45, *SE* = 1.07, *p* < 0.001) is twice the size of the Native group effect, in the opposite direction, meaning that Intermediate participants are significantly less accurate on semantically primed words vs. control trials.

In the pseudo priming trials at HF, the Native group (*b* = −1.17, *SE* = 0.45, *p* < 0.01) is significantly less accurate compared to control trials, and the Intermediate (*b* = −0.72, *SE* = 0.41, *p* = 0.08) and Advanced (*b* = −0.80, *SE* = 0.44, *p* = 0.07) groups are marginally showing an even stronger pseudo priming effect suggesting they are even less likely to answer those trials correctly.

For LF Control trials, Natives perform just as well as on HF trials. The Advanced group shows a frequency effect (*b* = −1.05, *SE* = 0.49, *p* = 0.03) in that they perform less well on LF control trials compared to the Native group. The Intermediate group was not exposed to LF trials due to proficiency limitations. The size of the semantic priming effect for LF trials is just as strong for Natives and Advanced as it is for HF trials, as the interaction terms indicate the semantic effect for LF trials is not significantly different than the effect for HF trials.

For pseudo priming LF trials, the effect for the Native group is the same as for HF trials, and the three-way positive interaction for the Advanced group (*b* = 1.18, *SE* = 0.58, *p* = 0.04) essentially negates the frequency effect. Put another way, the Advanced group does not show a pseudo priming effect for LF trials, but still performs marginally worse on pseudo prime trials compared to NSs as they did on HF trials. Interestingly, the Advanced and Native groups, despite obvious descriptive trends toward a frequency effect on pseudo priming trials, statistically show no frequency effect.

#### Reaction time

**Figure 2:**
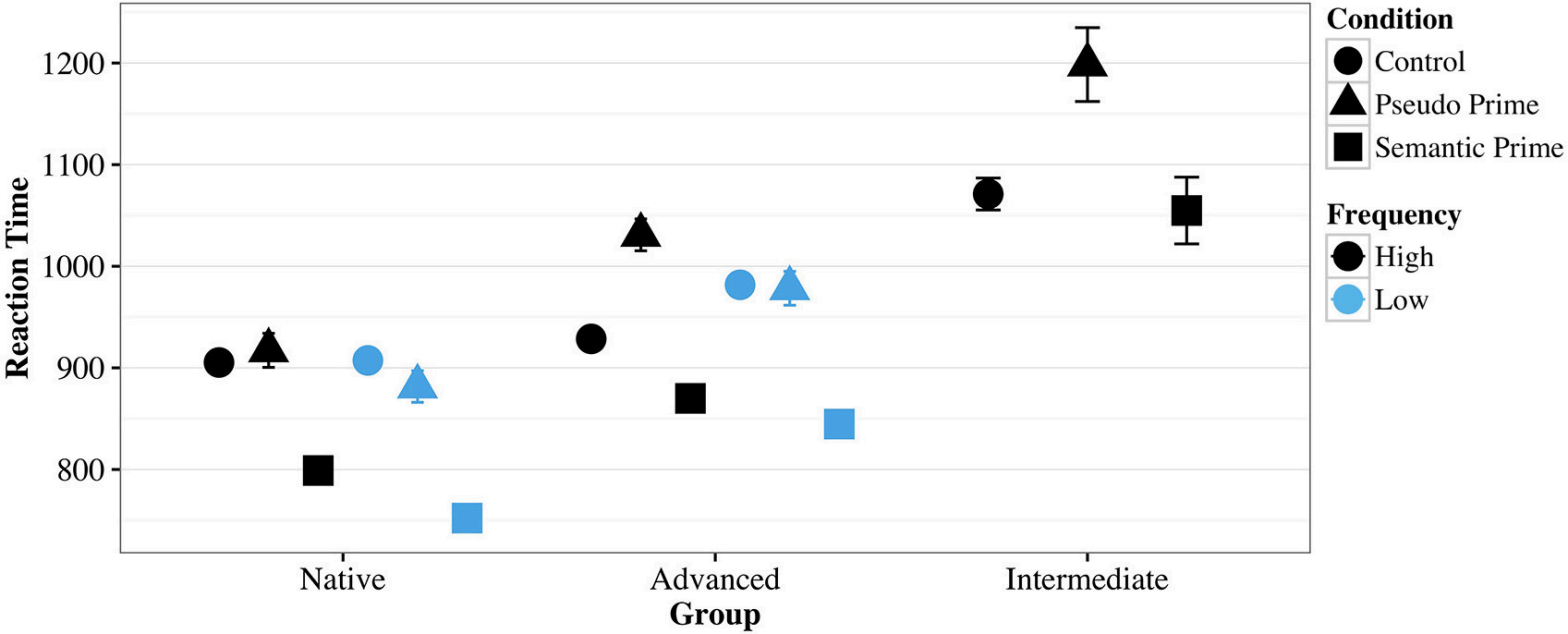
**Mean RTs of pseudo-semantic priming trials, semantic trials, and unrelated control trials split by language group for words of high and low frequency in Experiment 2**.

RT results for correct responses were trimmed as described previously (eliminating 1.8% of observations) and submitted to a linear multilevel model (Table 8). All fixed effects, including interactions and baselines were identical to those in the logistic MLM for the PSP accuracy data above. The random effects structure for the linear model differed again compared to the logistic MLM, again likely due to larger variability in RT compared to accuracy. To help visualize the effects presented in the model, a simplified characterization of the data as cell means is presented in Figure 2 (also see Supplemental Material for raw values).

**Table 8:** **Experiment 2 (PSP) results of linear multilevel modeling for RT**.

**Fixed effects**	***b***	***SE***	***t* value**
Intercept (Native/Control/HF)	6.79	0.03	228.64
Group:			
Intermediate	0.18	0.04	4.29^*^
Advanced	0.03	0.04	0.93
Condition:			
Semantic Priming (Native)	−0.12	0.02	−5.33^*^
Semantic × Intermediate	0.14	0.03	4.28^*^
Semantic × Advanced	0.06	0.02	2.38^*^
Pseudo Priming (Native)	0.01	0.02	0.57
Pseudo × Intermediate	0.04	0.03	1.25
Pseudo × Advanced	0.09	0.03	3.30^*^
Low frequency (Native)	0.004	0.02	0.20
LF × Advanced	0.05	0.02	2.40^*^
LF × Semantic (Native)	−0.06	0.03	−1.82^∧^
LF × Semantic × Advanced	−0.02	0.03	−0.70
LF × Pseudo (Native)	−0.04	0.03	−1.32
LF × Pseudo × Advanced	−0.06	0.04	−1.68^∧^
**Random effects**	**Variance**	***SD***	**Correlation**
Intercept | Subject	0.01	0.12	
Semantic | Subject	< 0.001	0.02	0.29
Pseudo | Subject	< 0.01	0.03	0.93 −0.04
Intercept | Prime	< 0.01	0.05	
Intermediate | Prime	< 0.01	0.05	−0.33
Advanced | Prime	< 0.001	0.02	−0.14 −0.88
Intercept | Prime/Item Pair	< 0.01	0.05
Intermediate | Prime/Item Pair	< 0.01	0.06	−0.59
Advanced | Prime/Item Pair	< 0.01	0.05	−0.40 0.97
Residual		0.02	0.15

* Significant at p < 0.05;

∧ *Marginal at p < 0.10*.

On HF Control trials, the Native and Advanced group (the latter not statistically different from the Native group) make correct judgments more quickly than the Intermediate group (*b* = 0.18, *SE* = 0.04, *t* = 4.29).

For HF semantic priming trials, the Native (*b* = −0.12, *SE* = 0.02, *t* = −5.33) and Advanced groups (*b* = 0.06, *SE* = 0.02, *t* = 2.38) show a significant semantic priming effect in that they are faster on semantic trials compared to controls; however, note the significant effect for the Advanced group indicates that the semantic priming effect for the Advanced group is half as strong as for the Native group. Finally, for the Intermediate group, the semantic priming effect is no longer observed (*b* = 0.14, *SE* = 0.03, *t* = 4.28), meaning the group shows no speedup or slowdown on semantically primed trials.

In the pseudo priming trials for HF, the Native group and the Intermediate group show no effect of pseudo priming compared to control trials. However, the Advanced group (*b* = 0.09, *SE* = 0.03, *t* = 3.30) responds significantly more slowly on pseudo priming trials.

For low frequency (LF) Control trials, Natives perform just as well as on HF trials. The Advanced group shows a frequency effect (*b* = 0.05, *SE* = 0.02, *t* = 2.40) in that they perform more slowly on LF control trials compared to the Native group. The Intermediate group was not exposed to LF trials. Compared to HF trials, the size of the semantic priming effect for LF trials shows a marginally larger effect for Natives (*b* = −0.06, *SE* = 0.03, *t* = −1.82) and Advanced (*b* = −0.02, *SE* = 0.03, *t* = −0.70), since the three-way interaction of LF × Semantic × Advanced was not significantly different from the marginal effect for Natives.

For pseudo priming LF trials, the Native group still shows no effect as for HF trials (*b* = −0.04, *SE* = 0.03, *t* = −1.32). The three-way marginal interaction for the Advanced group (*b* = −0.06, *SE* = 0.04, *t* = −1.68) indicates that (on top of the estimate and *t*-value for Natives) the Advanced group has no pseudo priming effect for LF trials (releveling the model with Advanced as baseline does exhibit this as a significant effect). Put another way, the Advanced group appears to only respond more slowly to HF pseudo priming trials and treats LF pseudo priming trials no differently than LF control trials.

### Discussion

Results of Experiment 2 primarily indicate that both Advanced learners and Native speakers were more likely to make an erroneous judgment on the target when it was primed by a word prime semantically-related to the competitor, such that when they heard *коРовА* /karova/ “cow” they were more likely to judge *молоток* /malatok/ “hammer” as a nonword compared to a similar sounding semantically related target *молоко* /malako/ “milk.” Consistent with our predictions, the Advanced learners show a processing delay with pseudo-semantic targets, albeit only in the high-frequency condition (we will come back to this point in the Section General Discussion). Unlike Advanced L2 learners, Native participants show no evidence of processing delays in either of the frequency conditions: they are as efficient in accessing a pseudo-semantic target as they are in accessing an unmatched control target. With the evidence of a robust semantic priming effect, we can conclude that no semantic priming effects guided their performance on pseudo-semantic targets.

The Intermediate group also shows no pseudo-semantic priming effect, but most likely for a different reason. We see here a similar trend as in the Advanced participants, but the slowdown is not supported statistically. The variability in the responses of the Intermediate participants is an indication that their lexical representations are unstable and are probably not yet sufficiently integrated into their mental lexicon. The conclusion is also supported by the lack of a semantic priming effect in the Intermediate group, which suggests that semantic associations among words in the developing L2 lexicon are not yet sufficiently entrenched to produce a nativelike semantic priming effect (for an entrenchment account, see Gollan et al., 2008; Diependaele et al., 2013; Cook and Gor, submitted).

Overall, the experiment has succeeded in demonstrating that even Advanced learners operate with fuzzy phonolexical representations, which do not ensure reliable access to the intended meaning of the input word. In the pseudo-semantic priming manipulation, the Advanced learners were biased toward a semantic associate of the prime. While Native speakers showed no evidence of engaging in the processing of a similar-sounding, but semantically unrelated competitor, the Advanced group did.

## General discussion

The results of the study are in line with the existing research in support of unfaithful L2 phonolexical representations (Pallier et al., 2001; Weber and Cutler, 2004; Darcy et al., 2012, 2013). Crucially, the present study extends the research agenda to demonstrate that the fuzziness of phonolexical representations can arise even when confusable words do not contain difficult phonological contrasts.

There is a current understanding that the acquisition of phonological categories and lexical representations, while being closely interrelated, still shows some autonomy in nonnative learners. This relative autonomy may lead to asymmetries between L2 efficiency in phonological encoding and lexical (or phonolexical) encoding (Weber and Cutler, 2004; Darcy et al., 2013). The present paper takes the next step in the direction of exploring the nature of L2 lexical encoding. It attempts to dissociate the L2 phonological and phonolexical encoding difficulties. It does so by focusing on similarly sounding L2 words that are not differentiated by difficult phonological contrasts (e.g., the hard-soft consonant contrast in Russian, as in Chrabaszcz and Gor, 2014).

In Experiment 1—the TJT—we looked at how Levenshtein Distance, which operationalizes the degree of phonolexical similarity between the words that are potential lexical competitors, affects native and nonnative lexical access. Native speakers do not show statistically significant sensitivity to phonolexical similarity between the target word and its implied competitor. This suggests that they have access to fully-specified, detailed phonolexical representations, which allow them to reliably reject words that are not a complete match to the stored representation and which is independent of the degree of phonological overlap. At the same time, as predicted, only the lower-proficiency speakers (Advanced group) show the effect of phonolexical similarity, which interacts with lexical frequency, and is much weakened in the higher-proficiency speakers (Superior group).

To challenge the processing delay interpretation that we are proposing in Experiment 1, one can hypothesize that the effect of phonolexical distance is due to the speedup in the words with greater phonolexical distance between the competitors instead. In following with this argument, the effect is driven not by the slowdown in the lexical access of words with a smaller phonological distance, but rather by faster access to the words with greater phonological distance. One can reject this interpretation based on the inspection of the results visually represented in Figure 1. When we compare the RTs in the LD and Unmatched conditions in the Advanced and Superior groups, it is clear that LD 1 and LD 2 items incur additional processing costs compared to the unmatched items, while LD 3 and LD 4 items do not. Therefore, low-similarity items in the Confusable condition were treated in the same way as the unrelated ones (this conclusion is also supported by the statistical analyses). In terms of the speed of access, the results for the Advanced and Superior groups in the control conditions, or for words without phonological overlap, are not significantly different from each other. These results provide us with reasonable grounds to claim that longer latencies in the processing of words with smaller phonological differences from the competitors are due to poorer quality of phonolexical representations, which entails a less efficient matching mechanism, and causes processing delays during lexical access in lower-proficiency L2 learners.

The performance of the Native participants in Experiment 1 also warrants additional discussion. It is typically assumed that native speakers of the language are more efficient and more rapid in performing lexical access than nonnative speakers (e.g., Gollan and Kroll, 2001; Michael and Gollan, 2005). The results of the present experiment do not challenge this observation, despite the fact that the RTs in the Native group are slower than in the two learner groups across all conditions. The experiment was designed as such that only the Russian primes can lead to a native processing advantage. However, the reaction times are measured on the responses to the English targets, which are the English translations of the Russian primes. It is quite reasonable to expect overall processing delays in the performance of the Native Russian group in processing of the English stimuli. The study explores how the relative difference in the processing speed of words with competitors varying in the degree of phonolexical similarity manifests itself in each individual group; therefore, the slowness in processing English stimuli of the native speakers does not interfere with the findings.

The result of Experiment 2—the Pseudo-Semantic Priming task—extends the finding of Experiment 1. Unlike native speakers, nonnative speakers do not properly encode phonolexical information, and, as indicated by the slowdown in the judgments on the confusable words, are thereby prone to access the incorrect lexical representation of a lexical competitor. Experiment 2 also succeeded in demonstrating that learners are unable to reliably access the word that they have heard because its phonolexical representation is not detailed enough, and are attempting to access the confusable word semantically related to the target instead.

This study provides evidence that nonnative ability to differentiate two lexical entries is not only affected by a perceptual inability to reliably identify L2 phonemes (as shown in other studies, e.g., Weber and Cutler, 2004; Cutler et al., 2006; Escudero et al., 2008; Hayes-Harb and Masuda, 2008), but is also associated with how well the word is known, or its degree of entrenchment in the mental lexicon (Diependaele et al., 2013; Veivo and Järvikivi, 2013; Cook and Gor, submitted). This conclusion is supported by the role of L2 learners' proficiency and lexical frequency during lexical access. The results of Experiment 1 demonstrate that at some point in the development of the L2 lexicon, learners operate with fuzzy phonolexical representations that lack detailed phonological encoding. With increasing proficiency, phonolexical representations acquire greater detail and become less fuzzy. This progression from fuzzy to fully detailed phonolexical representations is evidenced in how the sensitivity to the degree of mismatch between the auditory input and the existing representations affects lexical access in two nonnative groups with different proficiency levels and the native group. As the results of the experiment demonstrate, there is no delay in lexical access of the words that are different from their competitor only in one or two phonemes in native speakers. The higher-proficiency Superior group shows a tendency to some delay that does not reach statistical significance. Conversely, fuzzy representations preclude an effective match between the auditory input and the stored phonolexical representations, and thereby cause a significant slowdown in processing observed in the lower-proficiency nonnative group.

The effect of lexical frequency is observed in the results of Experiment 2, where only in the high-frequency condition did the Advanced learners show a processing delay in accessing the pseudo-semantic target. At Advanced proficiency, phonolexical representations of high-frequency words are sufficiently detailed, and the mismatch between the stimulus and the representation is readily detected; however, the ability to efficiently discount the competitor in favor of the correct target is not yet nativelike. The lack of a pseudo-semantic effect in the low-frequency condition suggests that the phonolexical form of these words in L2 does not have sufficient phonolexical detail to detect the mismatch, and thereby trigger a slowdown. Our findings are in full agreement with the entrenchment proposal based on a computer simulation that showed how lower levels of subjective familiarity lead to increased activation of such lexical entries and to a reduced ability to inhibit other activated candidates (Diependaele et al., 2010; see also Cook and Gor, submitted). These modeling results parallel recent empirical findings. Veivo and Järvikivi (2013) explored the role of L1 orthography in L2 lexical access of Finnish-French bilinguals and found that when a word did not have a stable L2 representation (as evidenced by subjective familiarity with the word), priming by an interlingual orthographic homophone resulted in a processing benefit attributable to prelexical facilitation. A similar conclusion in relation to phonolexical, rather than orthographic, representations was reached by Broersma (2012), who hypothesized that the lack of inhibition effect from error-induced homophones in a priming lexical decision for L2 learners (e.g., *flesh–flash*) should be taken as evidence for the reduced ability to mediate competition between the coactivated words.

Finally, our study provides evidence that fuzzy phonolexical representations result in unfaithful form-to-meaning mappings that lead to retrieval of incorrect semantic content. Experiment 2 has succeeded in establishing the effect of fuzzy form-meaning mappings on the activation of semantic networks during priming with a prime that was phonologically similar, but semantically unrelated to the target. The observed inhibition was due to spurious activation of a semantically plausible phonological neighbor. The results primarily suggest that the meaning of the competing words is not only activated, but considered as a possible meaning of the target. The involvement of the semantic level provides evidence in support of occurrence of erroneous form-to-meaning mappings in a developing L2 lexicon. As suggested by the *weaker links* hypothesis (Gollan et al., 2008) bilinguals split their language experience between two (or more) languages, and are, therefore, disadvantaged compared to monolingual speakers in terms of establishing lexical representations. Indeed, reduced exposure to nonnative words does not sufficiently strengthen the links between semantics and phonolexical representations. While our study is not designed to support or falsify the weaker links hypothesis, our results are compatible with it.

The present study provides further empirical evidence in support of the *fuzzy lexicon* hypothesis (Cook, 2012; Cook and Gor, 2015), which suggests that speed and accuracy of lexical access is mediated by the degree of detail in L2 phonolexical representations and by the strength of form-to-meaning mappings. In Experiment 2 we show that Advanced learners are sensitive to the semantic as opposed to pseudo-semantic priming manipulation. While they are able to detect the difference between the intended and the actual target, their difficulty lies in the ability to overcome the initial bias toward the semantic target. The processing slowdown indicates that the separation of phonolexical representations in the semantic and pseudo-semantic targets is not fully resolved, and lexical access of the correct meaning incurs additional processing costs. At the same time, the two representations are to a certain degree distinct from each other—a result also reported by Darcy et al. (2013). Had they been completely merged together, we would have observed a facilitation effect associated with semantic priming as an indication that the mismatching phonolexical form had activated the competitor's semantic meaning. This is not the mechanism that we observed. Instead, pseudo-semantic priming creates a semantic garden path that sets up a strong prediction, which is further confirmed by the target onset. This garden path effect is initially the same for both native and nonnative groups. While native participants quickly recover from this competition, with no additional processing costs observed, nonnative participants are not as efficient. On the one hand, they do not have sufficiently detailed representations to be certain about the match to the word they hear. On the other hand, they need to break the semantic connection from the prime to the pseudo-semantic target, to which they were guided by the prime, and further, by the initial phonological overlap of the actual target with the virtual semantic target. As both phonolexical and semantic representations are weak and generate uncertainty, the step of rejecting one lexical entry and reaccessing a different word (the pseudo-semantic target) incurs significant processing costs. The competition of form contributes to the processing costs, but it is mediated by a semantic link, and in this sense, the ambiguity resolution takes place at both levels—phonolexical and semantic.

Overall, the study takes the next step in identifying the locus of nonnative difficulties in lexical access and investigates challenges in phonological and lexical representations that go beyond discriminating difficult nonnative contrasts. It provides further evidence for the *fuzzy lexicon* hypothesis and empirically demonstrates that speed and accuracy of lexical access is mediated by the degree of detail in L2 phonolexical representations, which, in turn, is constrained by subjective familiarity with lexical items and L2 proficiency.

## Author contributions

SC and KG developed the theoretical framework, and designed the study. SC developed the materials and was in charge of data acquisition and writing the draft of the manuscript. NP contributed to the analysis and interpretation of the data, and drafting the Results Section. AL participated in data collection and drafting the Methods Sections of the paper. KG contributed to writing the Introduction, Present Study, and the Discussion Sections. SC, KG, NP, and AL reviewed the data, and contributed to certain aspects of the discussion. All authors are responsible for final approval of the manuscript, and endorse all aspects of its design, and the interpretation of the results.

### Conflict of interest statement

The authors declare that the research was conducted in the absence of any commercial or financial relationships that could be construed as a potential conflict of interest.

## References

[B1] Baayen H. (2008). Analyzing Linguistic Data: A Practical Introduction to Statistics Using R. Cambridge, UK: Cambridge University Press.

[B2] Baayen H. Davidson D. Bates D. (2008). Mixed-effects modeling with crossed random effects for subjects and items. J. Mem. Lang. 59, 390–412. 10.1016/j.jml.2007.12.005

[B3] Bates D. Maechler M. Bolker B. Walker S. (2015). lme4: Linear Mixed-Effects Models using Eigen and S4. R Package Version 1.1-9. Available online at: http://CRAN.Rproject.org/package=lme4

[B4] Beijering K. Gooskens C. Heeringa W. (2008). Predicting intelligibility and perceived linguistic distance by means of the Levenshtein algorithm, in Linguistics in the Netherlands, eds van Koppen M. Botma B. (Amsterdam: John Benjamins), 13–24.

[B5] Best C. T. (1994). The emergence of native-language infuence in infants: a perceptual assimilation model, in The Transition from Speech Sounds to Spoken Words: The Development of Speech Perception, eds Nusbaum H. Goodman J. Howard C. (Cambridge, MA: MIT Press), 167–224.

[B6] Best C. T. (1995). A direct realist view of cross-language speech perception, in Speech Perception and Linguistic Experience: Issues in Cross Language Research, ed Strange W. (Timonium, MD: York Press), 171–203.

[B7] Best C. T. McRoberts G. W. Goodell E. (2001). Discrimination of non-native consonant contrasts varying in perceptual assimilation to the listener's native phonological system. J. Acoust. Soc. Am. 109, 775–794. 10.1121/1.1332378 11248981 PMC2777975

[B8] Boersma P. Weenink D. (2013). Praat: Doing Phonetics by Computer (version 5.3. 56)[computer program]. Amsterdam.

[B9] Broersma M. (2012). Increased lexical activation and reduced competition in second-language listening. Lang. Cogn. Process. 27, 1205–1224. 10.1080/01690965.2012.660170

[B10] Broersma M. Cutler A. (2008). Phantom word activation in L2. System 36, 22–34. 10.1016/j.system.2007.11.00320737354

[B11] Broersma M. Cutler A. (2011). Competition dynamics of second-language listening. Q. J. Exp. Psychol. 64, 74–95. 10.1080/17470218.2010.499174 20737354

[B12] Chrabaszcz A. Gor K. (2014). Context effects in the processing of phonolexical ambiguity in L2. Lang. Learn. 64, 415–455. 10.1111/lang.12063

[B13] Conrad L. R. (1983). Listening Comprehension Strategies in Native and Second Language. Unpublished Doctoral dissertation, Michigan State University.

[B14] Cook S. (2012). Phonological Form in L2 Lexical Access: Friend or Foe? Unpublished Doctoral dissertation, University of Maryland.

[B15] Cook S. V. Gor K. (2015). Lexical access in L2: representational deficit or processing constraint? Ment. Lex. 10, 247–270. 10.1075/ml.10.2.04coo27291098

[B16] Cutler A. (2015). Representation of second language phonology. Appl. Psycholinguist. 36, 115–128. 10.1017/S0142716414000459

[B17] Cutler A. Otake T. (2004). Pseudo–homophony in non-native listening. J. Acoust. Soc. Am. 115, 2392 10.1121/1.4780547

[B18] Cutler A. Weber A. Otake T. (2006). Asymmetric mapping from phonetic to lexical representations in second-language listening. J. Phon. 34, 269–284. 10.1016/j.wocn.2005.06.002

[B19] Darcy I. Daidone D. Kojima C. (2013). Asymmetric lexical access and fuzzy lexical representations in second language learners. Ment. Lex. 8, 372–420. 10.1075/bct.80.06dar

[B20] Darcy I. Dekydtspotter L. Sprouse R. A. Glover J. Kaden C. McGuire M. (2012). Direct mapping of acoustics to phonology: on the lexical encoding of front rounded vowels in L1 English-L2 French acquisition. Second Lang. Res. 28, 5–40. 10.1177/0267658311423455

[B21] Davidson L. Shaw J. Adams T. (2007). The effect of word learning on the perception of non-native consonant sequences. J. Acoust. Soc. Am. 122, 3697–3709. 10.1121/1.2801548 18247777

[B22] Diaz B. Mitterer H. Broersma M. Sebastian-Galles N. (2012). Individual differences in late bilinguals' L2 phonological processes: from acoustic-phonetic analysis to lexical access. Learn. Individ. Differ. 22, 680–689. 10.1016/j.lindif.2012.05.005

[B23] Diependaele K. Lemhöfer K. Brysbaert M. (2013). The word frequency effect in first-and second-language word recognition: a lexical entrenchment account. Q. J. Exp. Psychol. 66, 843–863. 10.1080/17470218.2012.720994 23025801

[B24] Diependaele K. Ziegler J. C. Grainger J. (2010). Fast phonology and the bimodal interactive activation model. Eur. J. Cogn. Psychol. 22, 764–778. 10.1080/09541440902834782

[B25] Dufour S. Nguyen N. Frauenfelder U. H. (2010). Does training on a phonemic contrast absent in the listener's dialect influence word recognition? J. Acoust. Soc. Am. 128, EL43–EL48. 10.1121/1.3431102 20649188

[B26] Eckes T. Grotjahn R. (2006). A closer look at the construct validity of C-tests. Lang. Test. 23, 290–325. 10.1191/0265532206lt330oa

[B27] Escudero P. Hayes-Harb R. Mitterer H. (2008). Novel second-language words and asymmetric lexical access. J. Phon. 36, 345–360. 10.1016/j.wocn.2007.11.002

[B28] Fitzpatrick T. (2006). Habits and rabbits: words associations and the L2 lexicon. EUROSLA Yearbook 6, 121–145. 10.1075/eurosla.6.09fit

[B29] Flege J. E. (1995). Two procedures for training a novel second language phonetic contrast. Appl. Psycholinguist. 16, 425–442.

[B30] Flege J. E. Munro M. J. Fox R. A. (1994). Auditory and categorical effects on cross-language vowel perception. J. Acoust. Soc. Am. 95, 3623–3641. 10.1121/1.409931 8046152

[B31] Flege J. E. Takagi N. Mann V. (1996). Lexical familiarity and English-language experience affect Japanese adults' perception of /r/ and /l. J. Acoust. Soc. Am. 99, 1161–1173. 10.1121/1.414884 8609300

[B32] Forster K. I. Forster J. C. (2003). DMDX: a Windows display program with millisecond accuracy. Behav. Res. Methods Instrum. Comput. 35, 116–124. 10.3758/BF03195503 12723786

[B33] Gelman A. Hill J. (2007). Data Analysis using Regression and Multilevel/Hierarchical Models. New York, NY: Cambridge University Press.

[B34] Gollan T. H. Kroll J. F. (2001). Bilingual lexical access, in The Handbook of Cognitive Neuropsychology: What Deficits Reveal About the Human Mind, ed Rapp B. (New York, NY, US: Psychology Press), 321–345.

[B35] Gollan T. H. Montoya R. I. Cera C. Sandoval T. C. (2008). More use almost always means a smaller frequency effect: aging, bilingualism, and the weaker links hypothesis. J. Mem. Lang. 58, 787–814. 10.1016/j.jml.2007.07.001 19343088 PMC2409197

[B36] Gor K. (2014). Raspberry, not a car: context predictability and a phonological advantage in early and late learners' processing of speech in noise. Front. Psychol. 5:1449. 10.3389/fpsyg.2014.01449 25566130 PMC4271512

[B37] Gor K. (2015). Phonology and morphology in lexical processing, in The Cambridge Handbook of Bilingual Processing, ed Schwieter J. (Cambridge: Cambridge University Press), 173–199.

[B38] Goto H. (1971). Auditory perception by normal Japanese adults of the sounds “L” and “R”. Neuropsychologia 9, 317–323. 10.1017/cbo9781107447257.007 5149302

[B39] Hayes-Harb R. Masuda K. (2008). Development of the ability to lexically encode novel second language phonemic contrasts. Second Lang. Res. 24, 5–33. 10.1177/0267658307082980

[B40] Ingram J. C. Park S. G. (1998). Language, context, and speaker effects in the identification and discrimination of English /r/ and /l/ by Japanese and Korean listeners. J. Acoust. Soc. Am. 103, 1161–1174. 10.1121/1.421225 9479769

[B41] Kuhl P. K. Iverson P. (1995). Linguistic experience and the perceptual magnet effect, in Speech Perception and Linguistic Experience: Issues in Cross-Language Research, ed Strange W. (Baltimore, MD: York Press), 121–154.

[B42] Levenshtein V. I. (1966, February). Binary codes capable of correcting deletions, insertions, reversals. Soviet Physics Doklady 10, 707–710.

[B43] Linck J. A. Cunnings I. (2015). The utility and application of mixed-effects models in second language research. Lang. Learn. 65, 185–207. 10.1111/lang.12117

[B44] Luce P. A. Goldinger S. D. Auer E. T. Vitevitch M. S. (2000). Phonetic priming, neighborhood activation, and PARSYN. Percept. Psychophys. 62, 615–625. 10.3758/BF03212113 10909252

[B45] McClelland J. L. Thomas A. G. McCandliss B. D. Fiez J. A. (1999). Understanding failures of learning: hebbian learning, competition for representational space, and some preliminary experimental data. Prog. Brain Res. 121, 75–80. 10.1016/S0079-6123(08)63068-X 10551021

[B46] Meara P. (1983). Word associations in a foreign language: a report on the Birkbeck vocabulary project. Nottingham Linguist. Circ. 11, 29–38.

[B47] Meara P. (1984). The study of lexis in interlanguage, in Interlanguage, eds Davies A. Criper C. Howatt A. (Edinburgh: Edinburgh University Press), 225–235.

[B48] Meara P. M. (1978). Learners' word associations in French. Interlang. Stud. Bull. 3, 192–211.

[B49] Michael E. B. Gollan T. H. (2005). Being and becoming bilingual: Individual differences and consequences for language production, Handbook of Bilingualism: Psycholinguistic Approaches, eds Kroll J. F. de Groot A. M. B. (New York, NY: Oxford University Press), 389–407.

[B50] Niznik M. Vinokurova A. Voroncova I. Kagan O. Cherp A. (2009). Русский без границ [Russian without Borders]. Moscow: Russkij Mir.

[B51] Ota M. Hartsuiker R. J. Haywood S. L. (2009). The KEY to the ROCK: near-homophony in nonnative visual word recognition. Cognition 111, 263–269. 10.1016/j.cognition.2008.12.007 19230869

[B52] Pallier C. Bosch L. Sebastián-Gallés N. (1997). A limit on behavioral plasticity in speech perception. Cognition 64, B9–B17. 10.1016/S0010-0277(97)00030-9 9426508

[B53] Pallier C. Colomé A. Sebastián-Gallés N. (2001). The influence of native–language phonology on lexical access: exemplar–based versus abstract lexical entries. Psychol. Sci. 12, 445–449. 10.1111/1467-9280.00383 11760129

[B54] Pisoni D. B. Luce P. A. (1987). Acoustic-phonetic representations in word recognition. Cognition 25, 21–52. 10.1016/0010-0277(87)90003-5 3581727 PMC3514860

[B55] Polivanov E. (1932). The subjective nature of the perception of sounds. Polivanov 223–237.

[B56] R Core Team (2015). R: A Language and Environment for Statistical Computing. Vienna: R Foundation for Statistical Computing Available online at: http://www.R-project.org/

[B57] Reinisch E. Weber A. Mitterer H. (2013). Listeners retune phoneme categories across languages. J. Exp. Psychol. 39, 75–86. 10.1037/a0027979 22545600

[B58] Rüschemeyer S. A. Nojack A. Limbach M. (2008). A mouse with a roof? Effects of phonological neighbors on processing of words in sentences in a non-native language. Brain Lang. 104, 132–144. 10.1016/j.bandl.2007.01.004 17391746

[B59] Sekine S. (2006). The Effects of Phonological and Lexical Factors on L2 Word Recognition: A Cross-linguistic Study. Unpublished Doctoral dissertation, University of California Los Angeles, LosAngeles, CA.

[B60] Sheldon A. Strange W. (1982). The acquisition of /r/ and /l/ by Japanese learners of English: evidence that speech production can precede speech perception. Appl. Psycholinguist. 3, 243–261. 10.1017/S0142716400001417

[B61] Singleton D. (1999). Exploring the Second Language Mental Lexicon. Cambridge: Cambridge University Press.

[B62] Singleton D. Little D. (1991). The second language lexicon: some evidence from university-level learners of French and German. Second Lang. Res. 7, 62–81. 10.1177/026765839100700103

[B63] Singleton D. Singleton E. (1998). The C-test and L2 acquisition/processing research, in University Language Testing and the C-Test, ed Coleman J. A. (Portsmouth: University of Portsmouth), 150–178.

[B64] Stolz W. S. Tiffany J. (1972). The production of ‘child-like’ word associations by adults to unfamiliar adjectives. J. Verb. Learn. Learn. Behav. 11, 38–46. 10.1016/S0022-5371(72)80057-4

[B65] Strange W. (1995). Cross-language studies of speech perception: a historical review, in Speech Perception and Linguistic Experience: Issues in Cross-Language Research, ed Strange W. (Baltimore, MD: York), 3–48.

[B66] Veivo O. Järvikivi J. (2013). Proficiency modulates early orthographic and phonological processing in L2 spoken word recognition. Bilingualism 16, 864–883. 10.1017/S136672912000600

[B67] Weber A. Cutler A. (2004). Lexical competition in non-native spoken-word recognition. J. Mem. Lang. 50, 1–25. 10.1016/S0749-596X(03)00105-0

[B68] Wolter B. (2001). Comparing the L1 and L2 mental lexicon: a depth of individual word knowledge model. Stud. Second Lang. Acquis. 23, 41–69. 10.1017/S0272263101001024

[B69] Yarkoni T. Balota D. Yap M. (2008). Moving beyond Coltheart's N: a new measure of orthographic similarity. Psychon. Bull. Rev. 15, 971–979. 10.3758/PBR.15.5.971 18926991

