# Supplementary material for: Fuzzy Nonnative Phonolexical Representations Lead to Fuzzy Form-to-Meaning Mappings
Source: Front Psychol. 2016 Sep 21;7:1345. doi: 10.3389/fpsyg.2016.01345 (PMC5030242; doi:10.3389/fpsyg.2016.01345)
Supplement: Supplementary file 1 [file Table1.pdf]

Table 1: Mean raw reaction times (and standard deviations) to Russian match trials, non-competitor mismatch trials, and competitor trials of different Levenshtein distance split by frequency for both native and nonnative speakers in Experiment 1 (TJT). Smaller Levenshtein distance indicates greater phonological similarity.

Group	Frequency	Match	Non-Competitor Mismatch	Competitor Mismatch (Levenshtein Distance)			
				1	2	3	4
Native							
	High	666 (290)	806 (338)	854 (418)	834 (359)	761 (251)	779 (293)
	Low	718 (352)	781 (321)	792 (307)	808 (326)	832 (299)	797 (248)
Superior							
	High	640 (328)	727 (275)	867 (490)	809 (342)	740 (311)	720 (253)
	Low	671 (360)	717 (257)	793 (383)	819 (367)	792 (351)	798 (309)
Advanced							
	High	621 (324)	727 (267)	898 (485)	874 (366)	733 (312)	698 (201)
	Low	659 (328)	710 (245)	790 (366)	824 (393)	812 (356)	770 (259)
