# Supplementary material for: Fuzzy Nonnative Phonolexical Representations Lead to Fuzzy Form-to-Meaning Mappings
Source: Front Psychol. 2016 Sep 21;7:1345. doi: 10.3389/fpsyg.2016.01345 (PMC5030242; doi:10.3389/fpsyg.2016.01345)
Supplement: Supplementary file 2 [file Table2.pdf]

Table 2: Mean raw reaction time (and standard deviations) to Russian pseudo-semantic priming trials, semantic trials, and control trials split by frequency for both native and nonnative speakers in Experiment 2 (PSP). The Intermediate group was not exposed to low frequency trials.

Group	Frequency	Pseudo-Semantic Priming	Semantic Priming	Unrelated Control
Native				
	High	917<br>(220)	799<br>(171)	905<br>(189)
	Low	882<br>(194)	752<br>(143)	907<br>(183)
Advanced				
	High	1031<br>(229)	870<br>(159)	929<br>(161)
	Low	978<br>(232)	845<br>(168)	982<br>(200)
Intermediate				
	High	1198<br>(384)	1055<br>(348)	1071<br>(283)
